# Mygalin reduces inflammation by targeting TLR3 signaling pathway in macrophages

**DOI:** 10.3389/fimmu.2026.1729852

**Published:** 2026-06-12

**Authors:** Nayara Del Santos, Ricardo Vázquez-Ramírez, Abraham Espinoza-Culupú, Elizabeth Mendes, Mayara de Oliveira Cavalcante, Pedro Ismael Silva Junior, Monamaris Marques Borges

**Affiliations:** 1 Bacteriology Laboratory, Butantan Institute, São Paulo, Brazil; 2 Instituto de Investigaciones Biomédicas, Universidad Nacional Autónoma de México, Mexico City, Mexico; 3 Laboratory of Molecular Microbiology and Biotechnology, Faculty of Biological Sciences, Universidad Nacional Mayor de San Marcos, Lima, Peru; 4 Laboratory for Applied Toxinology (LETA), Butantan Institute, São Paulo, Brazil

**Keywords:** cell signaling, inflammation, innate immunity, molecular docking, molecular dynamics, Mygalin, RAW 264.7 macrophages, TLR3

## Abstract

Toll-like receptors (TLRs) are central regulators of innate immunity, enabling macrophages to detect pathological changes through both cell surface and intracellular compartments. Several molecules have been investigated for their ability to modulate TLR-mediated immune responses. Mygalin, a synthetic acylpolyamine analogue of spermidine, has been reported to modulate macrophage activation via extracellular TLR2 and TLR4 pathways. Here, we investigated the immunomodulatory effects of Mygalin on RAW 264.7 macrophages activated with Poly I:C (TLR3 agonist), LPS (TLR4 agonist), and IFN-γ using *in vitro* assays and *in silico* molecular docking. Mygalin treatment significantly attenuated the production of inflammatory mediators, reduced IRF3 phosphorylation without affecting STAT1, and selectively downregulated CD40 expression, while CD86, and F4/80 levels remained. No significant cytotoxicity was detected in any of the analyzed groups. Docking analyses revealed that Mygalin interacts with three distinct binding sites on TLR3, with the highest affinity for site 3, and engages specific residues of the IFN-γ receptor. Molecular dynamics simulations confirmed the stable association of Mygalin with TLR3 and the IFN-γ receptor. These findings suggest that Mygalin may represent a promising immunomodulatory candidate for controlling inflammatory conditions associated with dysregulated TLR signaling.

## 1 Introduction

Toll-like receptors (TLRs) constitute a family of pattern recognition receptors expressed by immune and non-immune cells that function as primary sensors of microbial and endogenous danger signals. By recognizing pathogen-associated molecular patterns (PAMPs) and danger-associated molecular patterns (DAMPs) released from stressed or damaged tissues, TLRs initiate innate immune responses and critically influence the development of adaptive immunity. Ligand engagement triggers intracellular signaling cascades that activate specific transcriptional programs, resulting in the production of pro-inflammatory cytokines and chemokines. These mediators coordinate immune cell recruitment, promote tissue repair, and shape antigen-specific immune responses (1). Although tightly regulated, TLR signaling must be precisely controlled, as dysregulated or excessive activation can drive pathological inflammation and contribute to autoimmune diseases, sepsis, and cancer (2, 3).

TLRs are broadly classified into cell surface and intracellular receptors according to their cellular localization and ligand specificity. Surface-expressed TLRs include TLR1 and TLR6, which heterodimerize with TLR2, as well as TLR4, TLR5, and TLR10. In contrast, intracellular TLRs–such as TLR3, TLR7, TLR8, and TLR9–are primarily localized to the endoplasmic reticulum and endosomal/lysosomal compartments, where they detect nucleic acids and other intracellular microbial components (1, 4).

TLR activation is controlled by multiple regulatory mechanisms that prevent sustained or excessive signaling. Upon ligand recognition, TLRs engage one of two major adaptor-dependent pathways: the MyD88-dependent pathway, utilized by all TLRs except TLR3, and the TRIF-dependent pathway, employed by TLR3 and TLR4. The MyD88 pathway predominantly promotes NF-κB activation and pro-inflammatory cytokine production, whereas the TRIF pathway is classically associated with the induction of type I interferon through activation of interferon regulatory factor 3 (IRF3) (3, 5). Despite this functional distinction, both pathways can contribute to inflammatory cytokine production depending on the cellular context and stimulus.

TLR3 recognizes viral double-stranded RNA (dsRNA) and synthetic analogs such as polyinosinic–polycytidylic acid (Poly I:C), triggering TRIF-dependent signaling and IRF3 activation (6). Although TLR3 activation is essential for antiviral defense, excessive or sustained TRIF–IRF3 signaling may contribute to pathological inflammation and tissue injury. In addition to promoting type I interferon responses, TLR3 activation in macrophages can drive the production of pro-inflammatory cytokines such as TNF-α and IL-6 in response to dsRNA stimulation, thereby amplifying inflammatory responses during viral infection (7, 8). Viral infections are frequently associated with acute or chronic inflammatory conditions that can be detrimental to the host if not properly controlled. Therefore, the identification of selective modulators capable of attenuating TLR3-driven inflammatory responses without broadly suppressing macrophage activation represents an important therapeutic objective.

Given their central role in pathogen sensing and immune activation, TLRs have emerged as attractive therapeutic targets. Several TLR agonists and antagonists are currently being investigated to modulate immune responses in infectious diseases, autoimmune disorders, and cancer (9, 10). However, strategies that selectively modulate downstream signaling pathways—particularly within the TLR3/TRIF axis—remain limited.

Mygalin is a synthetic acylpolyamine and structural analogue of spermidine, characterized by two terminal acyl groups and a molecular weight of 417 Da (11). Previous studies have demonstrated that Mygalin exhibits both microbicidal and immunomodulatory properties. *In vitro* and in silico analyses indicate that Mygalin interacts with the TLR4/MD2 complex, attenuating lipopolysaccharide (LPS)-induced inflammatory signaling in macrophages (12). Additional evidence suggests that Mygalin may also modulate membrane-associated TLRs, including TLR2/1 and TLR2/6 heterodimers, reinforcing its potential as an anti-inflammatory agent (13). However, its effects on intracellular TLRs, particularly TLR3, remain largely unexplored.

In the present study, we investigated the immunomodulatory effects of Mygalin on TLR3-driven inflammatory responses in RAW 264.7 macrophages stimulated with Poly I:C, LPS, and interferon-γ (IFN-γ). We assessed the production of inflammatory mediators, the activation of key transcription factors, the expression of surface molecules and cytotoxicity.

Additionally, molecular docking analyses were performed to explore potential interactions between Mygalin, TLR3, and the IFN-γ receptor, and molecular dynamics (MD) simulations to assess the stability of Mygalin binding to these targets. These findings identify Mygalin as a promising immunomodulatory candidate for controlling inflammation driven by dysregulated TLR signaling.

## 2 Materials and methods

### 2.1 Cell culture and treatments

The RAW 264.7 cell line is widely used to evaluate the immunomodulatory properties and cytotoxicity of new compounds, being the model chosen for the tests (14).

Cells were cultured in complete RPMI 1640 medium (Gibco, Invitrogen Corporation, Waltham, MA, USA) supplemented with 10% fetal bovine serum (FBS; Gibco, Invitrogen Corporation) and 25 µg/mL gentamicin. Cells were seeded at a density of 1 × 10^6^ cells in 500 µL per well in 48-well plates (Corning, New York, NY, USA) and incubated overnight at 37 °C in a humidified atmosphere containing 5% CO_2_. On the following day, cells were washed and resuspended in fresh RPMI medium prior to stimulation. RAW 264.7 cells were pretreated with Mygalin (90 or 360 µM) for 1 h, followed by stimulation with the TLR3 agonist polyinosinic:polycytidylic acid (Poly I:C, 5 µg/mL) or the TLR4 agonist lipopolysaccharide (LPS, 100 ng/mL), in the presence or absence of interferon-gamma (IFN-γ, 10 ng/mL). After 20 h of stimulation, culture supernatants were collected for analysis of immune mediators, including nitric oxide (NO), IL-6, and TNF-α. All agonists were purchased from InvivoGen (San Diego, CA, USA). LPS (100 ng/mL) was used as a positive control where indicated. RAW 264.7 cells were periodically tested for mycoplasma contamination to ensure cellular integrity.

Mygalin was synthesized using classical peptide chemistry methods (15), adapted as previously described by Pereira et al. (2017) (11) and Espinoza-Culupú (2020) (12). The compound was synthesized and purified at the Center for Research on Toxins, Immune Response and Cell Signaling (CeTICS—CEPID), Laboratory for Applied Toxinology (LETA), Butantan Institute, under the supervision of Dr. Pedro Ismael Silva Jr. After purification, the material was lyophilized, dissolved in endotoxin-free distilled water, and stored at −80 °C. For cell culture experiments, Mygalin was diluted in complete RPMI medium immediately prior to use.

### 2.2 Cell viability assay

RAW 264.7 cells (1 × 10^5^ cells/well) were seeded in 96-well plates and allowed to adhere overnight. Cells were then treated with Mygalin (0, 90 or 360 µM) for 20 h. Cell viability was assessed by MTT assay, [3-(4,5-dimethylthiazol-2-yl)-2,5-diphenyltetrazolium bromide]. Cell treated with 0.1% Triton X-100 were used as a positive control for cell death. After 4 h of incubation with MTT at 37 °C, the supernatant was carefully removed, and 100 µL of dimethyl sulfoxide (DMSO) was added to each well to dissolve the formazan crystals, followed by gentle agitation for 10 min. Absorbance was measured at 550 nm using a microplate reader (Multiskan EX, Thermo Fisher Scientific). Cell viability was expressed as a percentage relative to untreated control cells. Data are representative of two triplicate assays.

### 2.3 Measurement of nitric oxide and inflammatory cytokines

Nitric oxide (NO) production was indirectly determined by measuring nitrite (NO_2_^-^) accumulation in culture supernatants using the Griess reaction. Briefly, 50 µL of culture supernatant was mixed with 50 µL of 1% (w/v) sulfanilamide solution in a 96-well plate and incubated for 10 min at room temperature. Subsequently, 50 µL of 0.1% (w/v) N-(1-naphthyl) ethylenediamine dihydrochloride (NED) solution was added, followed by an additional 10 min incubation under the same conditions. Absorbance was measured at 550 nm using a microplate reader. Nitrite concentrations were determined from a standard curve generated with sodium nitrite (1–100 µM).

Tumor necrosis factor-alpha (TNF-α) and interleukin-6 (IL-6) levels were quantified in culture supernatants using commercial ELISA kits (TNF-α: cat. no. 88-7064-22; IL-6: cat. no. 88-7324-88; Invitrogen, Thermo Fisher Scientific, Inc., Waltham, MA, USA), according to the manufacturer’s instructions. Absorbance was measured at 450 nm using a Multiskan EX microplate reader (Thermo Fisher Scientific). Cytokine concentrations were calculated based on standard curves generated with recombinant cytokine standards. Samples were appropriately diluted to ensure values fell within the linear range of the assay. The detection ranges were 15.6–1,000 pg/mL for TNF-α and 8–500 pg/mL for IL-6.

All experiments were performed in three independent assays and conducted in triplicate.

### 2.4 Western blot analysis

RAW 264.7 macrophages were pretreated with Mygalin (90 or 360 µM) for 1 h and subsequently stimulated with Poly I:C (5 µg/mL) or LPS (100 ng/mL) for 6 h. After stimulation, cells were washed with ice-cold phosphate-buffered saline (PBS) and lysed in RIPA buffer (150 mM NaCl, 1% Triton X-100, 0.5% sodium deoxycholate, 0.1% SDS, 50 mM Tris-HCl, pH 8.0) supplemented with protease inhibitor cocktail (Sigma-Aldrich, St. Louis, MO, USA). Cell lysates were centrifuged at 14,000 rpm for 20 min at 4 °C, and the supernatants were collected for protein quantification using the Pierce™ BCA Protein Assay Kit (Thermo Fisher Scientific, Waltham, MA, USA), according to the manufacturer’s instructions. Equal amounts of protein (40 µg per lane) were separated by 10% SDS-PAGE and transferred onto nitrocellulose membranes (Bio-Rad Laboratories, Hercules, CA, USA). Membranes were blocked for 1 h at room temperature in Tris-buffered saline containing 0.1% Tween-20 (TBS-T) supplemented with 5% (w/v) bovine serum albumin (BSA). Membranes were then incubated overnight at 4 °C with the following primary antibodies: anti-phospho-IRF3 (Ser396) (cat. no. 4947), anti-phospho-STAT1 (S727) (cat. no. 9177), and anti-β-actin (cat. no. 4970). All antibodies used were diluted 1:1000 and purchased from Cell Signaling Technology (Cell Signaling Technology, Danvers, MA, USA). After washing with TBS-T, membranes were incubated for 1 h at room temperature with HRP-conjugated anti-rabbit IgG secondary antibody (cat. no.7074; dilution 1:2,000. Immunoreactive bands were detected using an enhanced chemiluminescence (ECL) detection system (Signaling Technology Inc. Denver, MA, USA) according to the manufacturer’s instructions, and visualized using an electronic documentation system (Uvitec, Cambridge, UK). Band intensities were quantified by densitometric analysis using ImageJ software and normalized to β-actin expression levels and expressed relative to the Poly I:C-treated group. All experiments were performed in at least three independent assays. Phosphorylation status is a well-established indicator of IRF3 and STAT1 activation during TLR signaling events; therefore, the analysis focused exclusively on phosphorylated proteins. The phosphorylated levels of IRF3 and STAT1 were normalized to β-actin, which served as a loading control to ensure equal protein input into the samples.

### 2.5 Flow cytometry analysis

For flow cytometry assays, Mygalin (300 µM) was selected based on prior cell viability analyses demonstrating the absence of cytotoxic effects at this concentration and its ability to modulate inflammatory responses under strong pro-inflammatory stimulation induced by Poly I:C and LPS plus IFN-γ.

The expression of surface markers CD40, CD86, and F4/80 on RAW 264.7 macrophages was evaluated. Cells (2 × 10^6^) were pretreated with Mygalin (300 µM) for 1 h and subsequently stimulated for 24 h with polyinosinic–polycytidylic acid (Poly I:C, 5 µg/mL) or lipopolysaccharide (LPS, 100 ng/mL), in the presence or absence of interferon-γ (IFN-γ, 10 ng/mL), at 37 °C in a humidified atmosphere containing 5% CO_2_. LPS plus IFN-γ was used as a positive control for classical (M1) macrophage activation. After treatment, cells were collected and washed twice with PBS (pH 7.4) containing 2% fetal bovine serum (FBS) at 4 °C. Fc receptors were blocked using unlabeled rat anti-mouse CD16/CD32 antibody (Fc block, cat. no. 14-0161-82) for 15 min on ice. Cells were then incubated for 1 h at 4 °C with fluorochrome-conjugated monoclonal antibodies: rat anti-mouse CD40-PE (cat. no. 12-0401-82), rat anti-mouse CD86-PE (cat. no. 12-0862-82), and rat anti-mouse F4/80-Tricolor (cat. no. MF48006). Appropriate matched isotype controls (rat IgG2a, κ) were used at the same concentrations. All antibodies were purchased from Invitrogen (Waltham, MA, USA). Staining was performed in PBS containing 2% FBS at 4 °C. After washing, cells were fixed in PBS containing 2% paraformaldehyde. Data acquisition was performed using a FACSCanto II flow cytometer (BD Biosciences), and at least 10,000 events were collected per sample. Macrophages were identified based on forward scatter (FSC) and side scatter (SSC) characteristics, followed by gating on singlet cells and the F4/80^+^ population. CD40^+^ and CD86^+^ expression was analyzed within F4/80^+^ cells. Data was expressed as mean fluorescence intensity (MFI) and percentage of positive cells and analyzed using FACSDiva software (version 9; BD Biosciences).

### 2.6 *In silico* docking analysis

#### 2.6.1 Interaction of Mygalin with IFN-γ receptor

Protein and Ligand Structures.

The three-dimensional (3D) structure of the interferon-gamma (IFN-γ) receptor complex was obtained from the Protein Data Bank (PDB ID: 6E3L) (16). All water molecules and non-essential chains not involved in the receptor–ligand interface were removed using Discovery Studio Visualizer (DSV) (17).

The 3D structure of Mygalin (C_21_H_27_N_3_O_6_) was retrieved from PubChem (CID: 57339218; https://pubchem.ncbi.nlm.nih.gov/compound/Mygalin).

#### 2.6.2 Docking analysis of Mygalin with IFN-γ receptor

The IFN-γ receptor complex (receptor) and Mygalin (ligand) were prepared for docking using AutoDock Tools (18). Hydrogen atoms were added, Gasteiger partial charges were assigned to all atoms, and non-polar hydrogens were merged. The docking grid was centered at coordinates x = −32.864, y = −15.601, z = 29.032, with a grid box size of 20 × 20 × 20 Å and a grid spacing of 0.375 Å. The exhaustiveness parameter was set to 8 to optimize the balance between computational efficiency and sampling accuracy.

Molecular docking simulations were performed using AutoDock Vina (19). The resulting binding poses were visualized and analyzed in PyMOL (20). Detailed interaction analysis was conducted using DSV, focusing on hydrogen bonding, hydrophobic interactions, and binding site compatibility to identify the key residues involved in the interaction between Mygalin and the IFN-γ receptor.

### 2.7 Interaction of Mygalin and poly I:C with TLR3

#### 2.7.1 Protein and ligand structures

The cryo-electron microscopy (cryo-EM) structure of TLR3 was obtained from the Protein Data Bank (PDB ID: 7WV3) for docking analysis. The complex consists of four TLR3 monomers (A, B, C, and D) bound to a synthetic RNA ligand (Poly I:C). For docking Mygalin with TLR3, water molecules, the Poly I:C, and monomers TLR3-A, TLR3-B, and TLR3-C were removed using Discovery Studio Visualizer (DSV) software. Interactions of Poly I:C with TLR3-D, as well as the interaction between TLR3-D and TLR3-A, were directly analyzed from the crystal structure to preserve biologically relevant binding interfaces.

#### 2.7.2 Docking analysis of Mygalin with TLR3

The TLR3-D monomer and Mygalin were prepared for docking analysis following the same procedure previously described for the IFN-γ receptor using AutoDock Tools. Docking simulations were performed between Mygalin and the TLR3-D monomer, sampling the entire surface of the monomer.

The docking grid was defined with center coordinates x = 179, y = 212, z = 117, and grid box dimensions x = 62, y = 82, z = 110 Å. All rotatable bonds of Mygalin were kept rigid with an exhaustiveness parameter set to 300. Molecular docking was performed using AutoDock Vina.

#### 2.7.3 Free energy of interaction of the TLR3-D-mygalin, TLR3-D-poly I:C and TLR3-D-TLR3-A

The free energy of interaction of the TLR3-D–Mygalin complex was calculated using the docking-derived structure presenting the most favorable binding energy. Residues involved in stabilizing the TLR3-D–Mygalin complex were identified using DSV. Only the first core of TLR3 residues belonging to the TLR3-Mygalin was considered for the calculations. Cutoff distances between the ligands and the receptor were set to 4.0 Å for hydrophobic interactions and 3.4 Å for hydrogen bonds.The free energy of interaction between Poly I:C and the TLR3-D monomer was calculated directly from the cryo-EM structure (PDB ID: 7WV3). TLR3-D residues interacting with Poly I:C at binding sites 1 (C-terminus) and 2 (N-terminus) were identified using the same structural criteria applied for the Mygalin to identify hydrophobic interaction and hydrogen bonds. Similarly, the interaction energy between TLR3-A and TLR3-D was calculated following the same procedure.

Interaction energy (IE) was determined using the equation: IE= [RL] + [R+L], where IE corresponds to the interaction energy of biding. RL is the energy of the complex formed by the receptor residues and the ligand, R is the energy of the TLR3-D residues, and L is the energy of the ligand. All binding interaction energies were calculated through single-point density functional theory (DFT) calculations using the ωB97X-D functional with the 6-31G* basis set, as implemented in Spartan’20 (21). For a detailed description of the *ab initio* methods employed, see reference (22). Docking and *ab initio* calculations were performed on a 16-core workstation equipped with an Intel Xeon processor operating at 3.4 GHz.

#### 2.7.4 Molecular dynamics simulations

To evaluate the stability of Mygalin binding to the IFN-γ receptor and TLR3, 50-ns molecular dynamics (MD) simulations were performed for both complexes using GROMACS 2025. Protonation states of ionizable residue were assigned at pH 7.2 using a PROPKA algorithm (23). The systems were solved using the TIP3P explicit water model (24) and neutralized with 0.15 M NaCl.

Energy system was minimized using the steepest descent algorithm and the Amber force field (25). Energy minimization was followed by NVT ensemble and NPT ensemble equilibration phases.

Long-range electrostatics interaction was calculated using Particle Mesh Ewald (PME) method (26) and bond constraints were applied using the LINCS (Linear Constraint Solver) algorithm. Trajectory stability was evaluated through the root mean square deviation (RMSD) and Principal Component analysis (PCA) (27).

### 2.8 Statistical analysis

All experiments were performed at least three independent times. Data was normalized. Data are presented as mean ± error of the mean SEM. Statistical analyses were conducted using GraphPad Prism (version 8). Comparisons between two groups were performed using Student’s *t*-test. For multiple group comparisons, one-way analysis of variance (ANOVA) followed by Tukey’s multiple comparison test was applied. Differences were considered statistically significant when p < 0.05.

## 3 Results

### 3.1 Mygalin reduces the inflammatory response induced by TLR3 activation

Previous studies have demonstrated that Mygalin modulates TLR2 and TLR4 signaling pathways (12, 13). To investigate whether Mygalin also affects TLR3-mediated activation, RAW 264.7 macrophages were stimulated with the TLR3 agonist Poly I: C.

**Figure 1:**
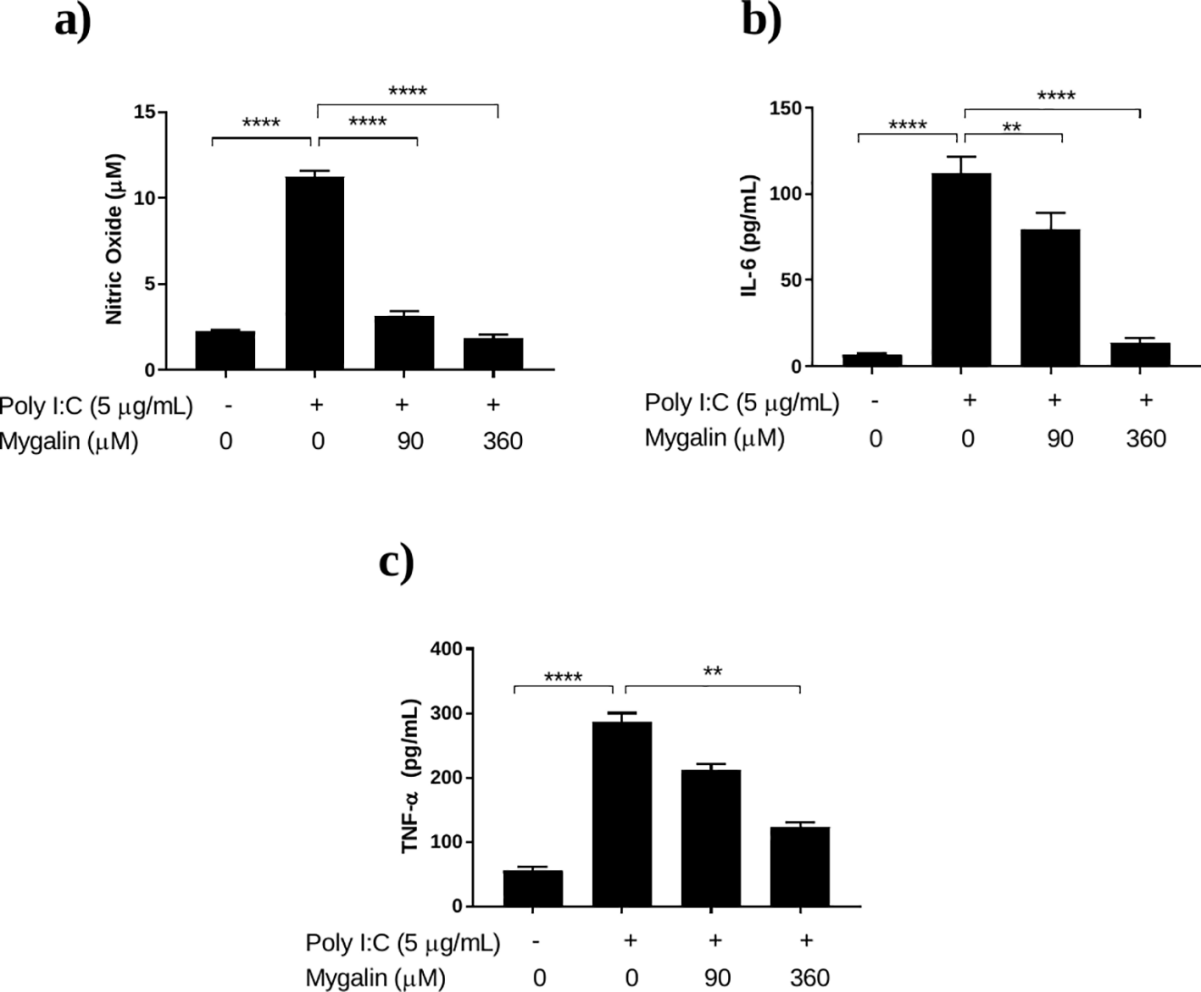
Mygalin Reduces the Inflammatory Response Induced by Poly I: C via TLR3 Activation. RAW 264.7 cells (1.0 × 10⁶/well) were pretreated with Mygalin (90 or 360 µM) for 1 h and then stimulated with Poly I:C (5µg/mL). After 20 h, culture supernatants were collected for quantification of nitric oxide (NO) using the Griess assay **(a)**, IL-6 **(b)** and TNF-α **(c)** by ELISA. Data are presented as mean ± SEM from three independent experiments. Statistical significance was determined by one-way ANOVA followed by Tukey’s multiple comparisons test. **p < 0.01; ****p < 0.0001.

Poly I:C stimulation markedly increased the production of nitric oxide (NO), interleukin-6 (IL-6), and tumor necrosis factor-alpha (TNF-α) compared with unstimulated cells. Pretreatment with Mygalin (90 or 360 µM) significantly reduced the production of all three inflammatory mediators following TLR3 activation. NO production was the most strongly affected parameter, showing reductions ranging from 72% to 83% compared with Poly I:C-stimulated cells, independently of Mygalin concentration. In contrast, significant inhibition of IL-6 and TNF-α production was observed predominantly at 360 µM, whereas 90 µM induced a modest reduction, particularly for TNF-α (approximately 29%). These findings indicate a dose-dependent effect of Mygalin on the production of inflammatory mediators (**Figure 1**).

### 3.2 Mygalin interferes with macrophage activation induced by IFN-γ, TLR3, and TLR4 agonists

To further investigate the effects of Mygalin on inflammatory mediator production, RAW 264.7 macrophages were stimulated with IFN-γ in combination with either Poly I:C (TLR3 agonist) or LPS (TLR4 agonist).

#### 3.2.1 Effect on the response induced by poly I:C and IFN-γ

**Figure 2:**
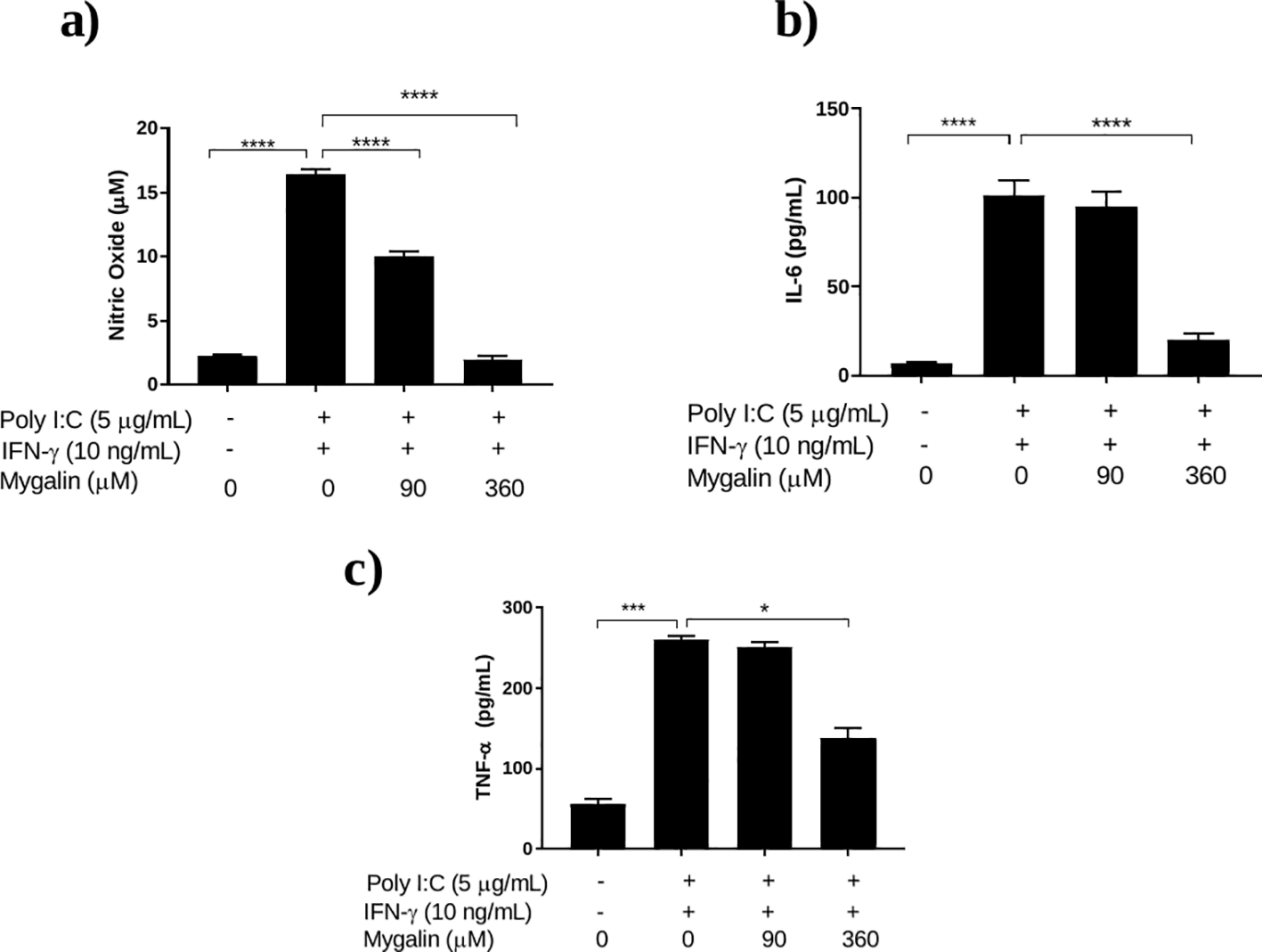
Mygalin attenuates the inflammatory response induced by Poly I:C + IFN-γ. RAW 264.7 cells (1 × 10⁶/well) were pretreated with Mygalin (90 or 360 µM) for 1 h and stimulated with Poly I:C (5 µg/mL) ± IFN-γ (10 ng/mL) for 20 h. Supernatants were analyzed for NO **(a)**, IL-6 **(b)**, and TNF-α **(c)**. Data are mean ± SEM of three independent experiments. Statistical significance was determined by one-way ANOVA followed by Tukey’s test. *p<0.05: ***p < 0.001; ****p < 0.0001.

RAW 264.7 macrophages stimulated with IFN-γ and poly(I:C) exhibited a robust inflammatory response, characterized by increased production of NO, IL-6, and TNF-α. However, pretreatment with Mygalin markedly reduced the levels of these mediators, particularly at 360 µM, resulting in reductions of 88% in NO, 80% in IL-6, and 47% in TNF-α production. In contrast, treatment with 90 µM Mygalin did not significantly alter IL-6 or TNF-α production compared with the stimulated control group (**Figure 2**).

#### 3.2.2 Effect on the response induced by LPS and IFN-γ

**Figure 3:**
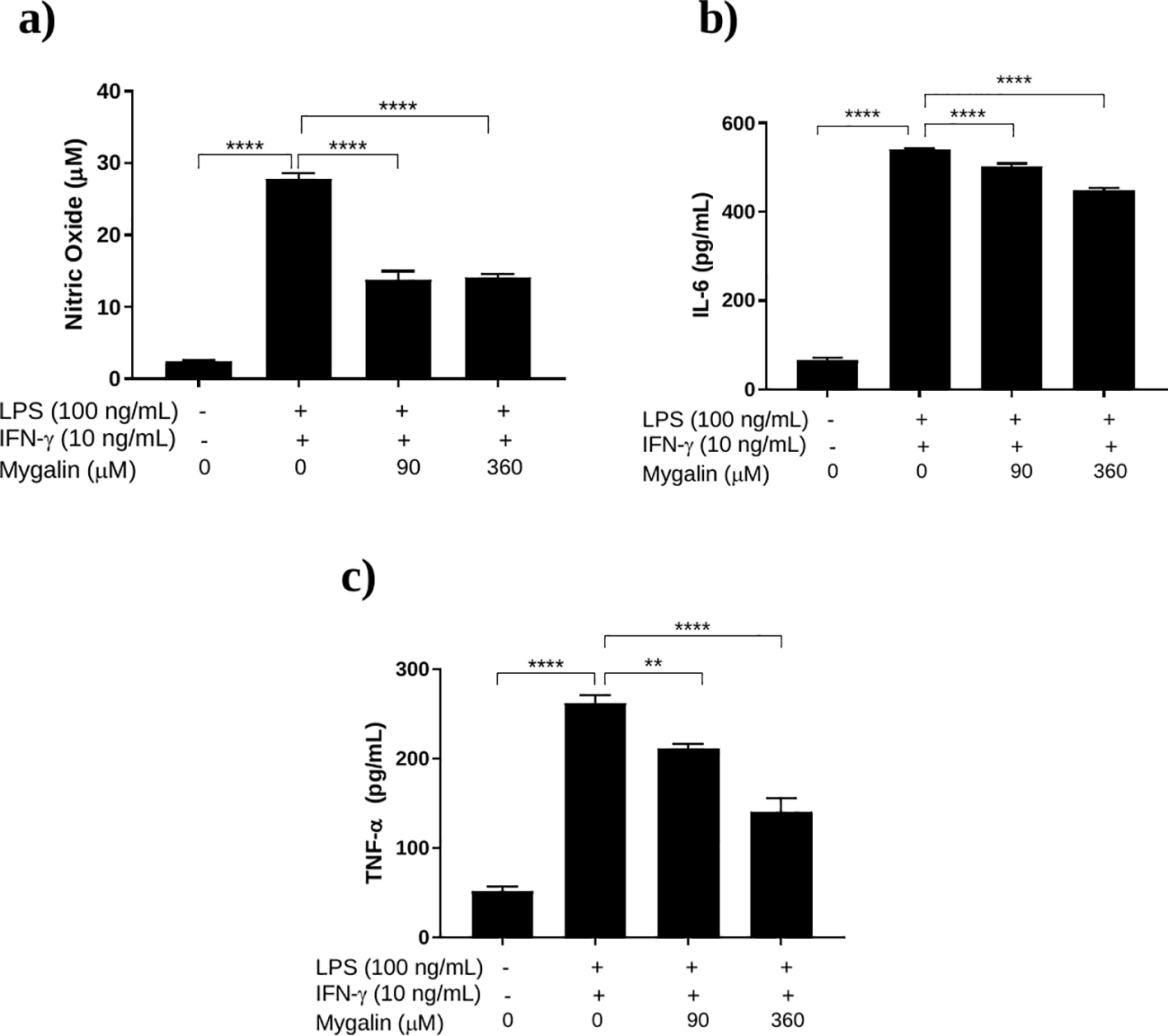
Mygalin modulates the inflammatory response induced by LPS + IFN-γ. RAW 264.7 cells (1 × 10⁶/well) were pretreated with Mygalin (90 or 360 µM) for 1 h and stimulated with LPS (100 ng/mL) ± IFN-γ (10 ng/mL) for 20 h. NO **(a)**, IL-6 **(b)**, and TNF-α **(c)** levels were measured. Data represent mean ± SEM of two independent experiments. Statistical significance was determined by one-way ANOVA with Tukey’s test. **p < 0.01; ****p < 0.0001.

Based on previous findings demonstrating that Mygalin reduces LPS-induced inflammation, we evaluated its effects on RAW 264.7 macrophages co-stimulated with IFN-γ and LPS. Pretreatment with Mygalin (90 or 360 µM) reduced the production of inflammatory mediators, resulting in approximately 50% inhibition of NO, 19–46% reduction in TNF-α, and 17% reduction in IL-6 levels. A similar modulatory pattern was observed in macrophages stimulated with IFN-γ and poly(I:C), (**Figure 3**).

### 3.3 Influence of Mygalin on the expression of surface molecules in phagocytic cells activated by TLR3 and TLR4 Agonists Plus IFN-γ

To evaluate whether Mygalin modulates the expression of surface molecules, RAW 264.7 macrophages were stimulated with poly(I:C) or LPS, in the presence or absence of IFN-γ, and the surface expression of CD40, CD86, and F4/80 was analyzed.

**Figure 4:**
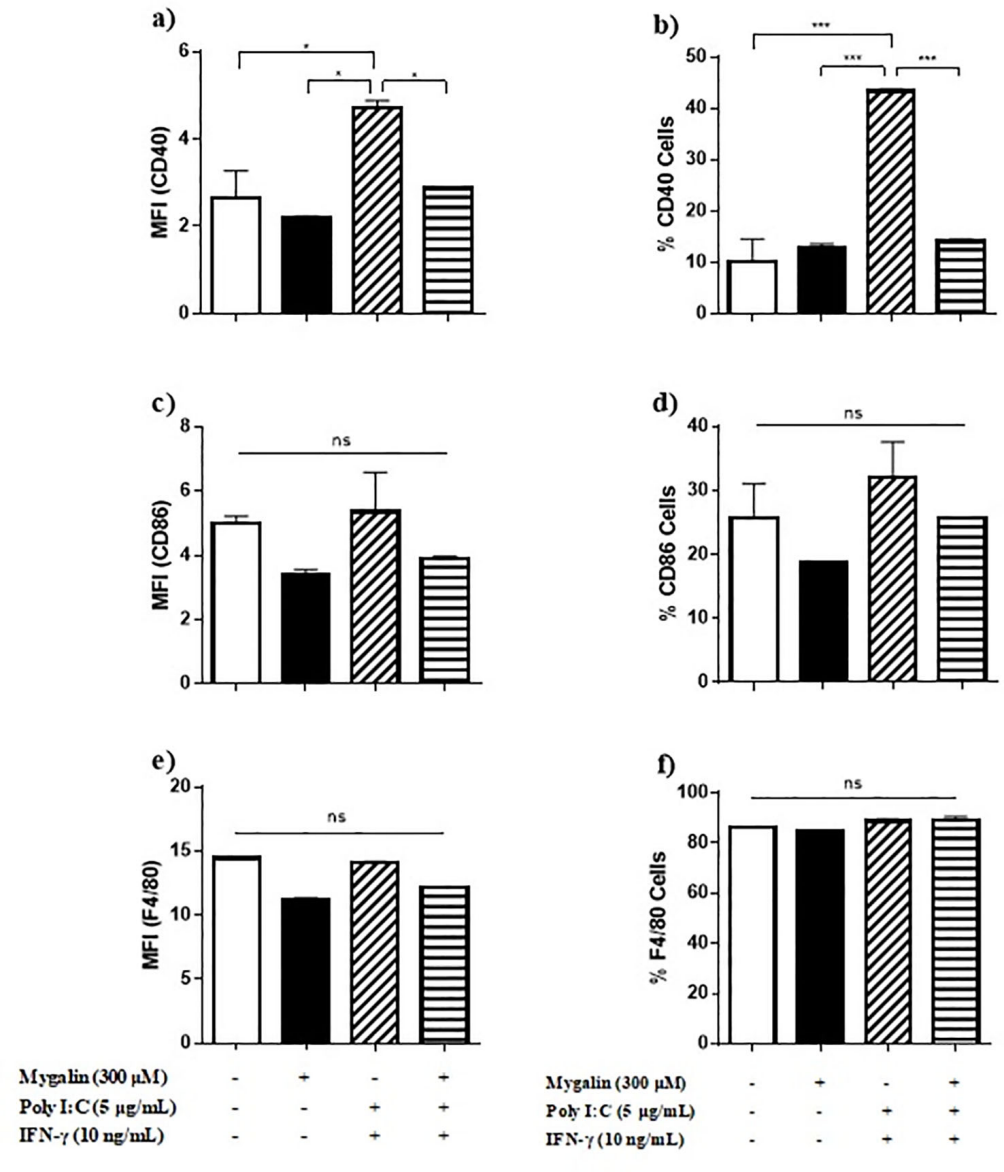
Effect of Mygalin on the expression of CD40, CD86, and F4/80 in macrophages activated with Poly I:C ± IFN-γ. RAW 264.7 cells (1 × 10^6^/well) were pretreated with Mygalin (300 µM) for 1 h and stimulated with Poly I:C (5 µg/mL) ± IFN-γ (10 ng/mL) for 24 h. Cells were labeled with antibodies against CD40, CD86, and F4/80 and analyzed by flow cytometry. Data are presented as mean fluorescence intensity (MFI) for CD40 **(a)**, CD86 **(c)**, F4/80 **(e)** and percentage of CD40+/F4/80+ **(b)**, CD86+/F4/80+ **(d)**, and F4/80+ cells **(f)**. F4/80 was used as a macrophage identity marker. Statistical significance was determined by one-way ANOVA followed by Tukey's test. *p < 0.05; ***p < 0.001.

**Figure 5:**
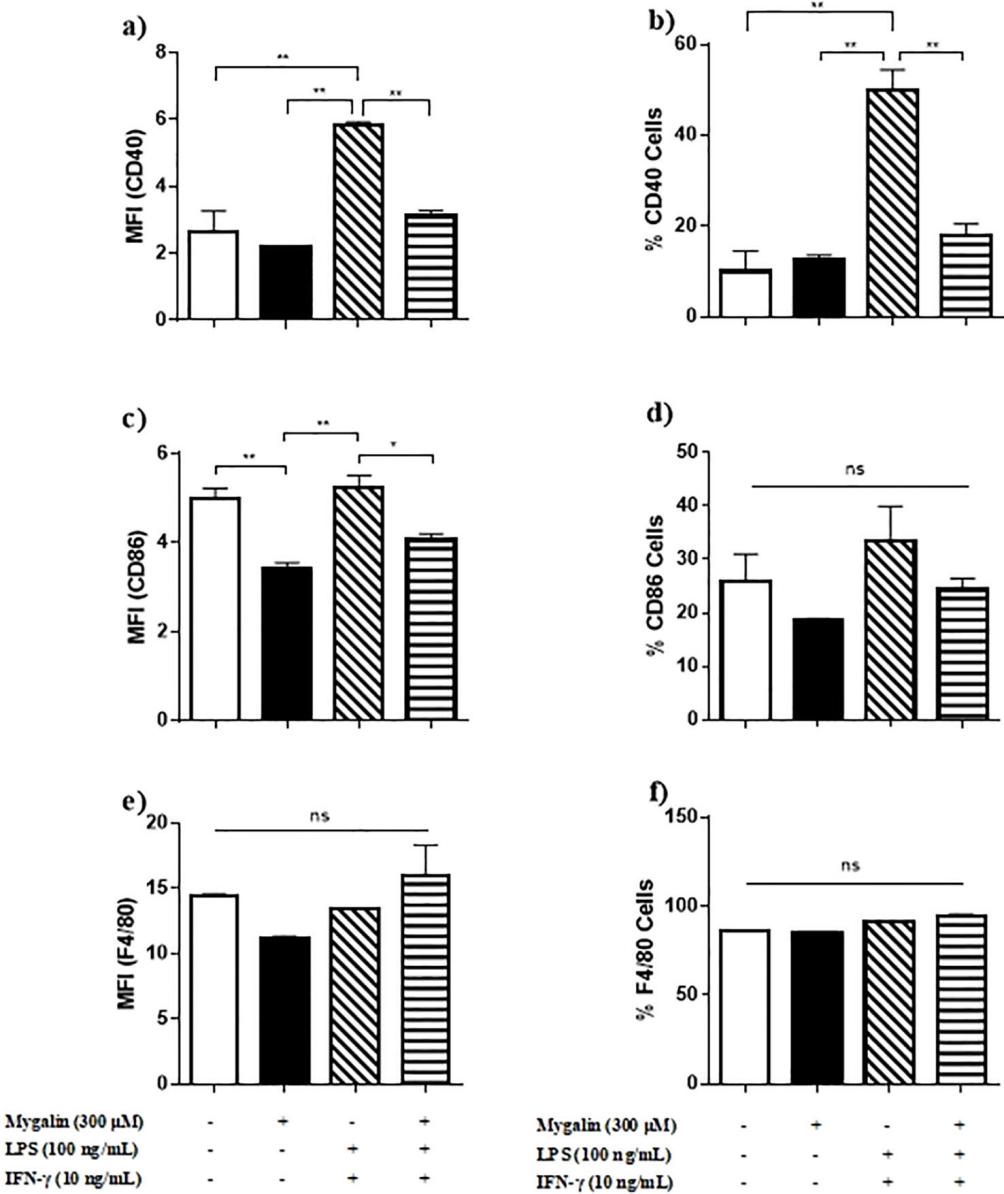
Effect of Mygalin on the expression of CD40, CD86, and F4/80 in macrophages activated with LPS ± IFN-γ. RAW 264.7 cells (1 × 10^6^/well) were pretreated with Mygalin (300 µM) for 1 h and stimulated with LPS (100 ng/mL) ± IFN-γ (10 ng/mL) for 24 h. Cells were stained for CD40, CD86, and F4/80 and analyzed by flow cytometry. Data are presented as MFI for CD40 **(a)**, CD86 **(c)**, F4/80 **(e)** and percentage of CD40+/F4/80+ **(b)**, CD86+/F4/80+ **(d)**, and F4/80+ cells **(f)**. F4/80 served as a macrophage marker. Statistical significance was determined by one-way ANOVA followed by Tukey's test. *p < 0.05; **p < 0.01; ***p < 0.001.

Stimulation with poly(I:C) (**Figure 4**) or LPS plus IFN-γ (**Figure 5**) increased the frequency of CD40^+^F4/80^+^ cells following TLR3- and TLR4-mediated activation. Pretreatment with Mygalin reduced both the mean fluorescence intensity (MFI) and the percentage of CD40^+^F4/80^+^ cells to levels comparable to those of unstimulated controls (**Figures 4A**, **B**, **5A**, **B**). In contrast, CD86^+^F4/80^+^ expression (**Figures 4C**, **D**, **5C** ,**D**) was not significantly altered by any treatment. Moreover, treatment with Mygalin alone did not affect the expression of any of the analyzed markers under the experimental conditions tested. The macrophage marker F4/80 remained unchanged across all groups, indicating that Mygalin did not alter macrophage phenotype under these conditions.

The effect of Mygalin on the expression of surface molecules in macrophages stimulated with LPS and IFN-γ was analyzed (**Figure 5**). As expected, co-stimulation with LPS and IFN-γ increased the frequency of CD40^+^F4/80^+^ cells in RAW 264.7 macrophages, whereas CD86^+^F4/80^+^ and total F4/80^+^ levels remained unchanged compared to untreated controls. Pretreatment with Mygalin (360 µM) significantly reduced both the mean fluorescence intensity (MFI) and the percentage of CD40^+^F4/80^+^ cells to levels comparable to unstimulated controls (**Figures 5A**, **B**). In contrast, Mygalin selectively reduced the MFI of CD86^+^F4/80^+^ cells without affecting the frequency of CD86^+^F4/80^+^ cells or the total F4/80^+^ population, indicating no impact on basal marker expression. These findings indicate that Mygalin selectively downregulates CD40 expression in response to inflammatory stimuli, independent of whether the activating receptor is surface-localized (TLR4) or intracellular (TLR3), and regardless of co-stimulation with IFN-γ.

### 3.4 Mygalin reduces IRF3 Phosphorylation without Altering STAT1 expression during TLR3 activation

The effect of Mygalin on intracellular signaling downstream of TLR activation was evaluated by analyzing the phosphorylation status of IRF3 and STAT1. Because activation of these pathways is primarily regulated through phosphorylation-dependent mechanisms, the analysis focused exclusively on the phosphorylated forms of these transcription factors.

**Figure 6:**
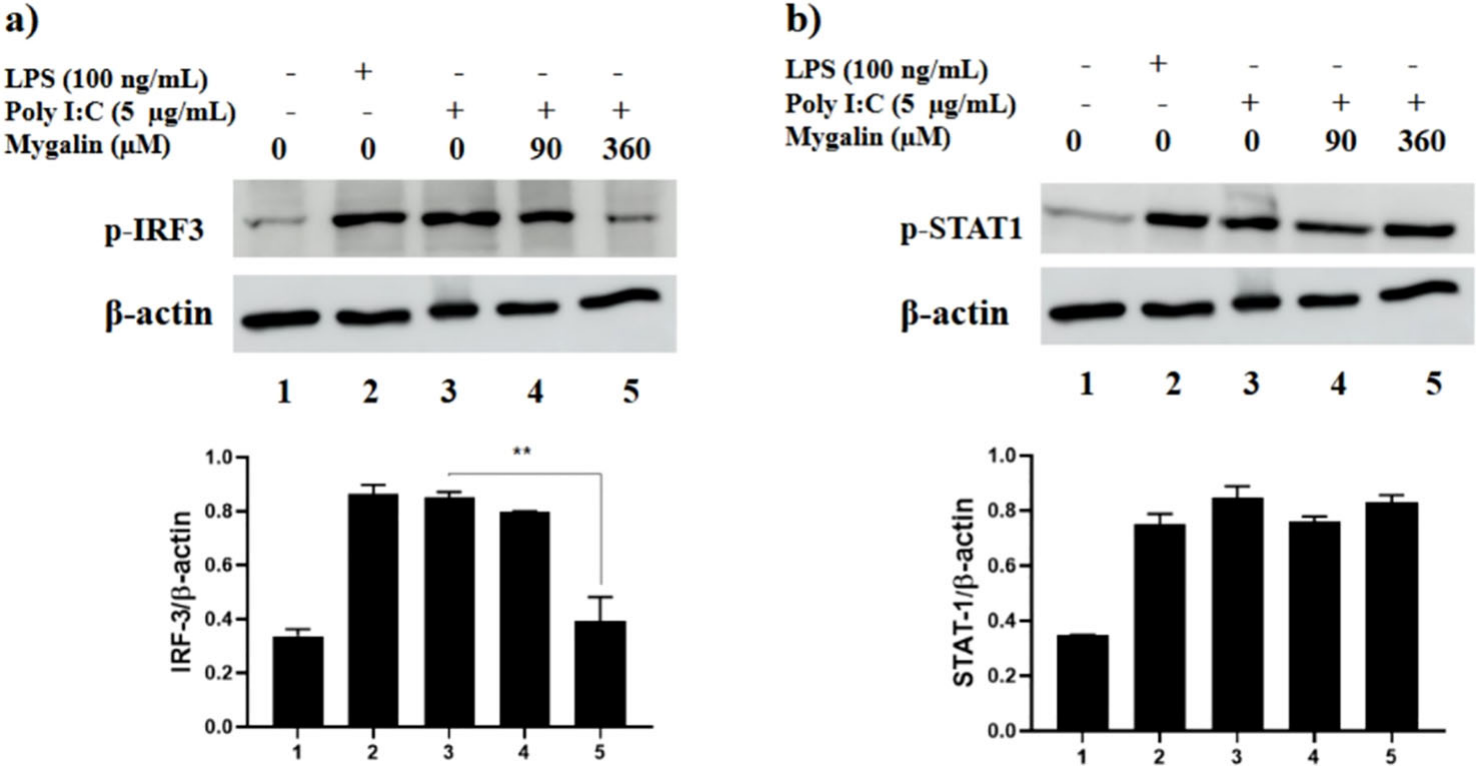
Mygalin reduces IRF3 phosphorylation without altering STAT1 phosphorylation. Western blot analysis of RAW 264.7 cells pretreated with Mygalin (0, 90, 360 µM) and stimulated with Poly I:C (5 µg/mL) for 20 h. LPS (100 ng/mL) was used as a positive control. Protein bands for p-IRF3 **(a)** and p-STAT1 **(b)** were quantified by densitometry and normalized to β-actin. Data are expressed relative to the Poly I:C-treated group and represent mean ± SEM of three independent experiments. Statistical significance was determined by one-way ANOVA followed by Tukey’s test. *p < 0.05.

As shown in **Figure 6**, stimulation with poly(I:C) or LPS increased the phosphorylation of both IRF3 and STAT1, confirming activation of TLR3 and TLR4-dependent signaling pathways. Pretreatment with Mygalin (360 µM) significantly reduced IRF3 phosphorylation, whereas STAT1 phosphorylation remained unaffected. Treatment with 90 µM Mygalin did not alter the phosphorylation of either molecule under the experimental conditions tested. These findings indicate that high-dose Mygalin selectively modulates IRF3 activation, a key regulator of type I interferon and pro-inflammatory gene expression, without broadly suppressing downstream transcription factors such as STAT1. These data suggest that Mygalin selectively modulates the TLR3–IRF3 signaling pathway, contributing to regulation of the inflammatory response.

### 3.5 Viability of RAW 264.7 cells treated with Mygalin, poly I:C, LPS or IFN-γ.

**Figure 7:**
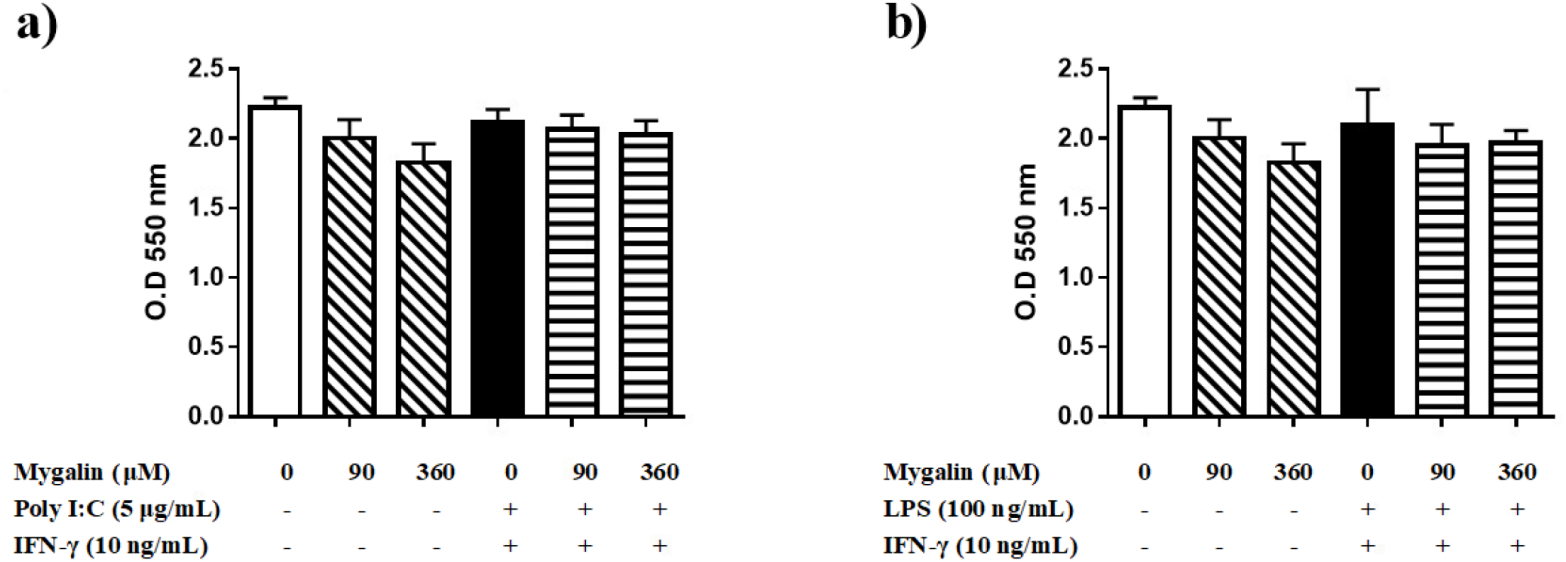
Mygalin does not affect RAW 264.7 cell viability. RAW 264.7 cells (1 × 10^6^/well) were pretreated with Mygalin (90 or 360 µM) for 1 h and stimulated with Poly I:C (5 µg/mL), LPS (100 ng/mL), or IFN-γ (10 ng/mL) for 20 h. Cell viability was assessed using the MTT assay, and optical density was measured at 550 nm. Viability **(a, b)** was expressed relative to untreated control cells (100% viable). Triton X-100 -treated cultures served as a positive control for cell death. Bars represent mean ± SEM of three independent experiments.

To exclude potential cytotoxic effects of Mygalin under the experimental conditions, RAW 264.7 macrophages were incubated for 20 h with increasing concentrations of Mygalin (0, 90, 360 µM). As shown in **Figure 7**, Mygalin did not affect cell viability at any concentration tested. In contrast, treatment with Triton X-100 resulted in a 100% reduction in viability, confirming the sensitivity of the assay. These results indicate that the immunomodulatory effects observed in the experimental assays are not attributable to cytotoxicity.

## 4 *In silico* analyses

### 4.1 Interaction of Mygalin with IFN-γ receptor

**Figure 8:**
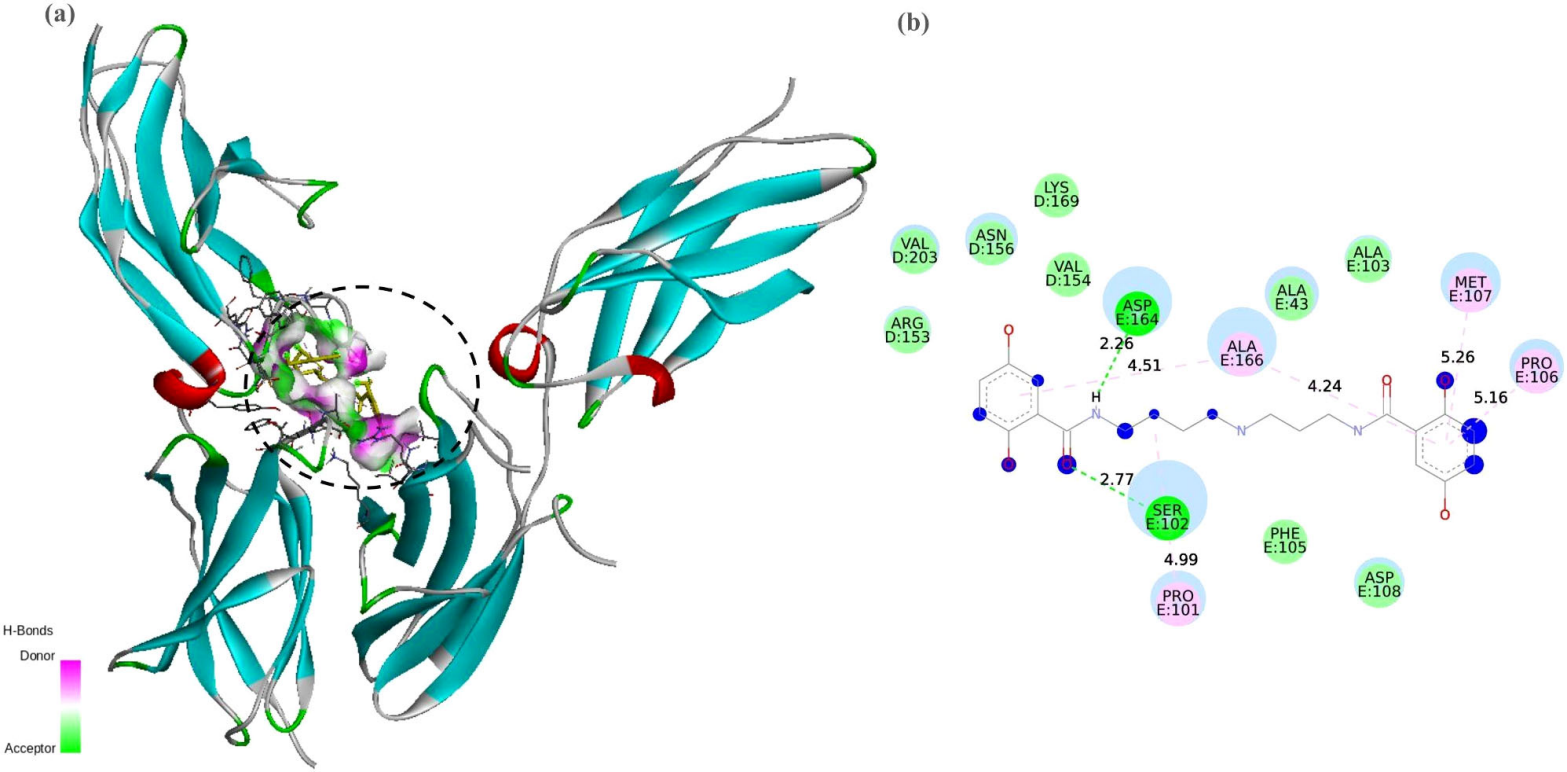
Molecular interaction between Mygalin and the interferon receptor. **(a)** Three-dimensional representation of the Mygalin–receptor complex. The ligand (yellow) is positioned within the binding site of the interferon receptor. Hydrogen bond interactions are indicated, with donor atoms shown in magenta and acceptor atoms in green. **(b)** Two-dimensional schematic showing amino acid residues involved in ligand binding. Key interactions include contacts with Asp164, Ser102, Pro101, and Ala166, among others. The predicted binding free energy for the complex was −26.78 kJ/mol, suggesting a stable and thermodynamically favorable interaction between Mygalin and the interferon receptor.

Molecular docking analysis showed that Mygalin binds to a defined region of the interferon-γ receptor, forming multiple hydrogen bonds and hydrophobic interactions that stabilize its position within the binding site. The three-dimensional representation, (**Figures 8A**, **B**) shows Mygalin nestled between the receptor’s β-sheets, establishing key contacts with residues Asp164, Ser102, and Ala166. The predicted binding free energy was −26.78 kJ/mol, suggesting a moderate yet thermodynamically favorable interaction. These findings suggest that Mygalin may interfere with IFN-γ-induced signaling, which is consistent with the effects observed on macrophage inflammatory responses in our experiments.

### 4.2 Interaction between TLR3 and Poly I:C complexes

**Figure 9:**
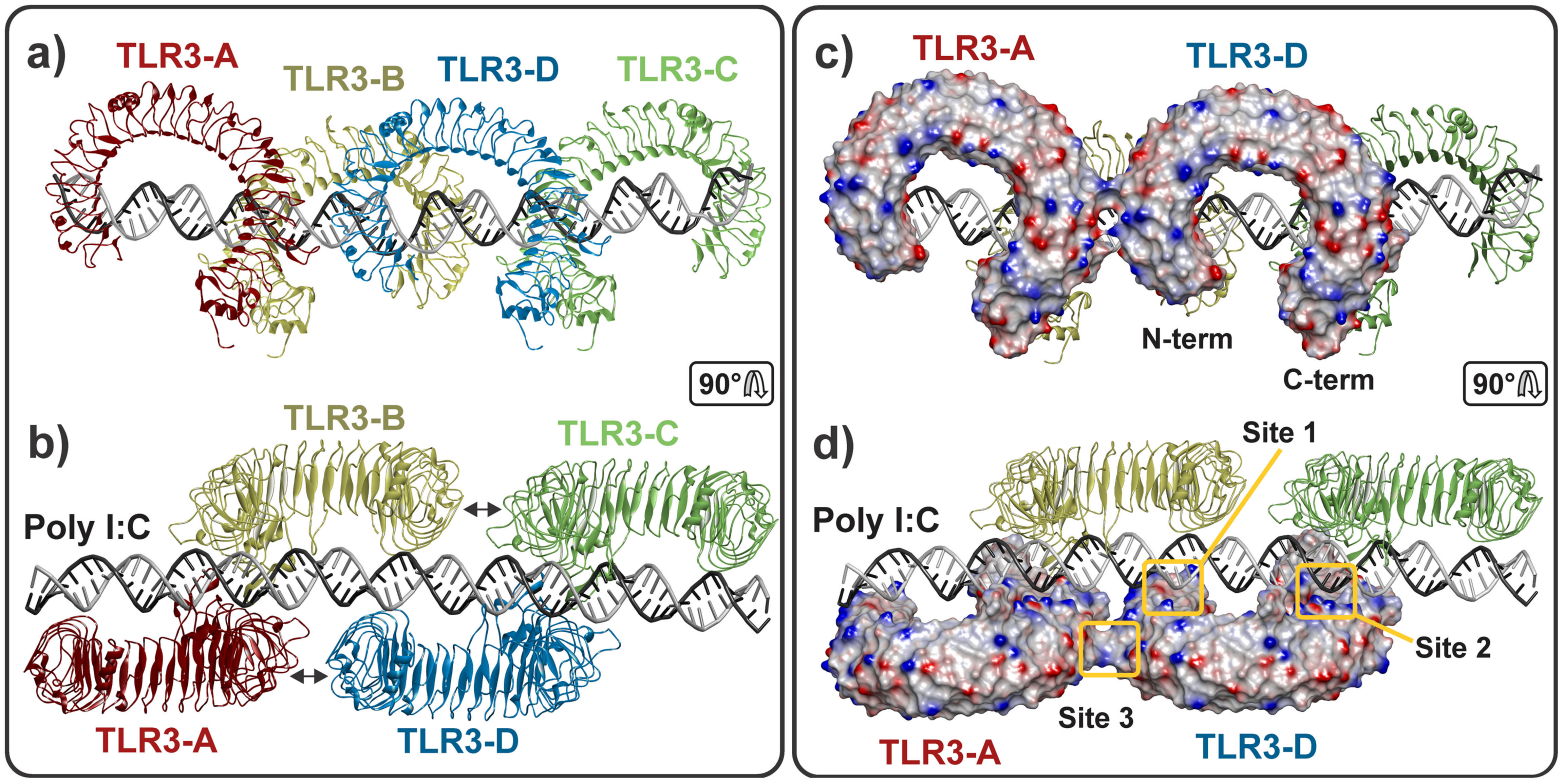
Interaction of TLR3 with Poly I: C Crystal structure of two tandem dimers, the first established between TLR3-A and TLR3-B, and the second between TLR3-C and TLR3-D complexed with a large (80bp) segment of Poly I;C, in frontal **(a)** and longitudinal **(b)** views. **(c)** The electrostatic potential is shown only in TLR3-A and TLR3-D, revealing a small interaction interface between the two monomers. **(d)** The squares indicate the two canonical Poly I:C binding sites and site 3 between the two monomers in the longitudinal view.

The *in silico* analysis was based on the three-dimensional X-ray crystallographic structure of the TLR3–dsRNA complex. It has been reported (28) have demonstrated that the oligonucleotide chain must have a minimum length of 40–50 base pairs RNA in order to dimerize TLR3 and initiate downstream signaling. More recent studies (29) report that the Poly I:C in crystallographic complex with TLR3 can involve at least 80 base pairs, and form clusters of two TLR3 homodimers in a longitudinal tandem configuration (**Figure 9**). The molecular docking analysis of Mygalin will be compared with the structural of Poly I:C, a synthetic double-stranded RNA analog that act via TLR3 leading to a potent immune response characterized by the production of inflammatory cytokines, including IL-1β, IL-6, TNF-α, as well as type I interferons (30, 31).

#### 4.2.1 Ligand–TLR3 interaction

Poly I:C interacts with TLR3-D through two canonical binding sites of interaction on TLR3-D with some differences: site 1, is close to the N-terminus (N-term) of TLR3-D and has an interaction interface of 582.4 Å². Site 2 is close to the C-terminus (C-term) and is slightly smaller at 572.1 Å². A third interaction site was considered for TLR3-D, which is located on the outer curvature, close to the N-terminus, and has an interaction surface of 53.1 Å² with TLR3-A. The residues involved in the interaction interface of the three sites are summarized in **Supplementary Table 1** and illustrated in **Figure 9D**. Docking analysis of Mygalin with TLR3-D revealed stable complex at sites 1 and 2 canonical for dsRNA were obtained. It also had docking at site 3, where the interdimer interface is located, as indicated in **Table 1** (**Supplementary Material**).

**Table 1:** TLR3-D binding sites.

TLR3-D site 1	Poly I:C	Interaction	TLR3-D site 2	Poly I:C	Interaction
His39	inosinic	H bond	Arg489	cytidylic	ionic
Lys41	inosinic	H bond	Asn515	cytidylic	ionic
His60	inosinic	ionic	Asn517	cytidylic	H bond
Asn61	inosinic	H bond	Ala519	inosinic	hydrophobic
Gln62	inosinic	H bond	His539	cytidylic	ionic
Arg64	cytidylic	H bond	Asn540	cytidylic	H bond
Arg65	cytidylic	ionic	Asn541	cytidylic	H bond
Phe84	inosinic	H bond	Arg544	inosinic	H bond
Thr86	cytidylic	H bond / hydrophobic	Ser571	cytidylic	H bond
Ser88	cytidylic	H bond	Gly573	cytidylic	H bond
Lys89	cytidylic	ionic	Lys619	inosinic	H bond
Lys108	inosinic	H bond			

TLR3-D sitie 3	Mygalin atom (group)	Interaction	TLR3-D site 3	TLR3-A	Interaction
Ser115	C (acyl 1)	hydrophobic	Ser115	Asn522	polar
Asp116	OH (acyl 1)	H Bond	Asp116	Ser498	polar
Asp116	NH (polyamine)	H Bond	Lys117	Asp523	polar
Asp116	C (polyamine)	hydrophobic	Lys117	Asp524	polar
Asp116	OH (acyl 2)	H Bond	Lys139	Ser498	polar
Lys117	OH (acyl 1)	H Bond	Asn140	Pro499	hydrophobic
Lys117	C (acyl 1)	hydrophobic	Asn141	Arg473	polar
Asn140	C (polyamine)	hydrophobic	Lys145	Glu527	polar
Asn141	O (polyamine)	H Bond	Thr166	Arg473	hydrophobic
Asn141	C (acyl 2)	hydrophobic	Gln167	Arg47	polar
Val144	OH (acyl 2)	H Bond	Gln167	Gln503	polar
Val144	C (acyl 2)	hydrophobic			
Lys145	OH (acyl 2)	H Bond			
Lys145	C (acyl 2)	hydrophobic			
Gln167	O (polyamine)	H Bond			

Sites 1 and 2: interaction with Poly I:C. Site 3: interaction with Mygalin and interface between TLR3-D and TLR3-A.

### 4.3 Interaction of TLR3 with Mygalin

#### Free energy of interaction

**Figure 10:**
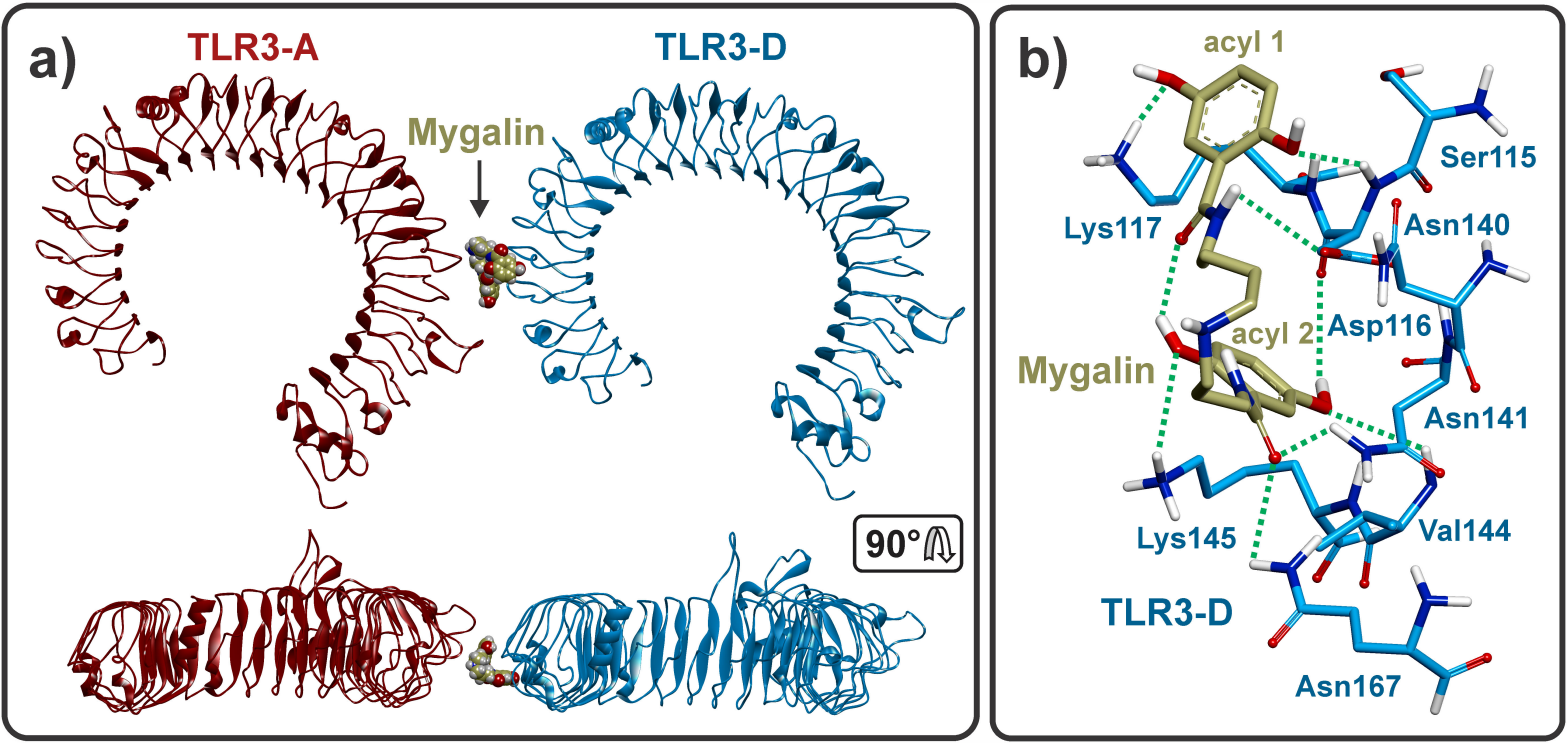
Interaction of TLR3 with Mygalin. Molecular docking analysis of the TLR3-Mygalin complex. **(a)** Mygalin binds to site 3 on the TLR3-D monomer; the complex is shown in both frontal and longitudinal views. **(b)** The residues interaction and hydrogen bonds (dotted lines) involved in the interaction with Mygalin are shown.

The binding of Poly I:C was especially high at both sites interaction with TLR3-D due to the occurrence of ionic interactions. Site 1 exhibited a free energy of −2,672.60 kJ/mol, while site 2 showed −2,850.62 kJ/mol, indicating that both sites contribute −5,523.22 kJ/mol per monomer. On the other hand, the interface formed between TLR3-D and TLR3-A monomers (site 3), had a free energy of −598.93 kJ/mol. Docking calculations for the Mygalin–TLR3-D complexes resulting from the free energies of −298.25 kJ/mol at site 1 and −375.28 kJ/mol at site 2, site 3 displayed the most favorable binding at −449.21 kJ/mol (**Figure 10**).

### 4.4 Molecular dynamics supports the stability of Mygalin–receptor complexes

MD simulations confirmed the stability of Mygalin binding to both IFN-γ receptor and TLR3. RMSD analysis revealed an initial equilibration phase followed by stable trajectories, with conformational stabilization occurring after 22.5 ns for the Mygalin–IFN-γ complex and 11 ns for the Mygalin–TLR3 complex. During the stable phase, RMSD values remained below 1.5 Å, indicating minimal conformational deviation from the docked structures. PCA analysis identified key residues contributing to binding stability. In the IFN-γ receptor complex, Asp164 emerged as a central stabilizing residue. In the TLR3 complex, residues Asn140, Asn141, Lys145, Ser115, Lys117, and Asp116 exhibited the highest interaction frequencies. These findings corroborate the docking predictions and support the persistence of Mygalin at the proposed binding sites (**Supplementary Figures 1**, **2**).

## 5 Discussion

This study demonstrates that Mygalin exerts a significant anti-inflammatory effect by modulating TLR3-directed signaling in macrophages. Mygalin significantly reduced the production of NO, IL-6, and TNF-α in RAW 264.7 macrophages stimulated with Poly I:C alone or in combination with IFN-γ, as well as with LPS plus IFN-γ, particularly at 360 µM. This effect was dose-dependent and not attributable to cytotoxicity, as confirmed by MTT viability assays.

Previous studies using RAW 264.7 and J774A.1 macrophage cell lines demonstrated that Mygalin, at concentrations ranging from 60 to 2,000 µM, did not induce cytotoxicity or alter basal protein expression profiles. Moreover, bone marrow-derived macrophages (BMDM) and J774A.1 cells treated with Mygalin in the presence of LPS exhibited reduced NO and TNF-α production compared to cells stimulated with LPS alone, further supporting the suppressive capacity of this molecule across different macrophage models. In those studies, *in silico* virtual screening analyses demonstrated Mygalin binding to the MD2 component of the TLR4/MD2 complex, suggesting a structural mechanism capable of modifying inflammatory signaling (12).

A similar modulatory effect was observed in J774 cells stimulated with TLR2/1 (Pam3CSK4) and TLR2/6 (Zymosan) agonists, indicating that Mygalin-mediated regulation is not exclusively dependent on MD2 interaction (13). Our data demonstrates that Mygalin also modulates intracellular TLR3-mediated responses induced by Poly I: C. Because TLR3 is localized within endosomal compartments and signals through the TRIF-dependent pathway, these results suggest that Mygalin can interfere with intracellular receptor signaling mechanisms, extending its immunomodulatory activity beyond surface TLR modulation.

Surface marker analysis revealed that Mygalin selectively decreases CD40+/F4/80+ expression in response to TLR3 and TLR4 stimulation, whereas CD86+/F4/80+ and total F4/80+ populations remain unchanged. These results indicate a targeted immunomodulatory effect while preserving basal macrophage identity. CD40 downregulation may help limit excessive pro-inflammatory responses triggered by TLR3 and TLR4 without compromising antigen presentation and basal costimulatory capacity. By modulating CD40 and IRF3 without affecting STAT1, Mygalin can limit excessive inflammatory processes induced by TLR3 signaling in several diseases (32–34).

Regarding intracellular signaling pathways using Raw 264.7 macrophages as a model, Mygalin at 360 µM significantly reduced IRF3 phosphorylation during TLR3 activation induced by Poly I:C, without affecting STAT1 phosphorylation. IRF3 is activated via TRIF and functions as a central regulator of type I interferon production and pro-inflammatory gene expression, whereas STAT1 phosphorylation occurs through the canonical JAK/STAT pathway following cytokine receptor engagement (34).

These findings indicate that Mygalin can limit excessive activation of a specific inflammatory signaling pathway without inhibiting others, acting more as an immunomodulator than a broad suppressor. These transcription factors operate at distinct and complementary stages of the innate immune response during macrophage activation through TLRs (33).

A comparable pattern of selective modulation was observed in J774A.1 macrophages treated with Mygalin and stimulated with a TLR2/1 agonist, where reduced activation of NF-κB p65 and STAT1 was detected (13). The differential impact on STAT1 activation during TLR3 versus TLR4 stimulation may be attributed to features of the signaling pathways (TRIF versus MyD88), receptor localization (endosomal versus plasma membrane), and the structural features of the respective agonists.

Future work should explore additional signaling pathways to delineate Mygalin’s mechanism of action. *In vivo* validation of its immunomodulatory effects and safety profile will also be critical for its application. molecule.

The *in silico* analysis of Mygalin interaction with the IFN-γ receptor revealed moderate affinity, particularly involving residues Asp164, Ser102, and Ala166, supporting the hypothesis that Mygalin may interfere with IFN-γ signaling. The predicted thermodynamically favorable binding and hydrogen bond formation suggests stable receptor engagement, consistent with its immunomodulatory effects. These findings align with previous observations of Mygalin interactions with Toll-like receptor complexes, including TLR2 and TLR4 (12, 13). Given that IFN-γ signaling drives iNOS expression and nitric oxide production in macrophages, direct binding of Mygalin to the interferon receptor suggests an additional mechanism contributing to its anti-inflammatory activity, supporting a broader modulatory role at concentrations above 150 µM.

Docking analyses further indicated that Mygalin binds to three sites on TLR3. Sites 1 and 2 correspond to canonical dsRNA-binding regions (28, 29), whereas site 3 is located in the convex region near the N-terminus, a region implicated in TLR3 dimer clustering (29). Although Mygalin exhibited favorable interaction energies at sites 1 and 2, these values were markedly lower than those observed for Poly I:C, suggesting that Mygalin is unlikely to competitively displace the ligand. Instead, the high affinity of Poly I:C for TLR3 and the dependence of receptor activation on dsRNA length and multimerization (34, 35) support an alternative mechanism. The localization of Mygalin at site 3, with interaction energy comparable to that of TLR3 dimer interfaces, suggests that it may interfere with receptor clustering or cooperative signaling rather than ligand binding, which is consistent with the attenuation of TLR3-driven responses observed *in vitro*.

The molecular dynamics simulations further strengthen the docking findings by demonstrating the temporal stability of Mygalin at the proposed binding sites. The low RMSD values and persistent interactions with charged residues, particularly Asp164 in the IFN-γ receptor and Lys145/Asp116 in TLR3, suggest that electrostatic interactions play a central role in stabilizing the complexes. Notably, the stability observed at the TLR3 interdimer interface supports the hypothesis that Mygalin may interfere with receptor clustering rather than directly competing with dsRNA binding, providing a mechanistic basis for its selective immunomodulatory effect on TLR3-driven inflammatory signaling.

Rather than broadly suppressing macrophage activation, Mygalin appears to recalibrate signaling intensity and pathway balance. Together, the structural predictions and cellular responses support a model in which Mygalin fine-tunes inflammatory signaling dynamics through context-dependent receptor engagement.

Taken together, the data highlights the potential of Mygalin as a selective modulator of innate immune signaling pathways and supports further *in vitro* and *in vivo* studies to define its potential application in diseases involving chronic inflammation.

## Data Availability

The original contributions presented in the study are included in the article/**Supplementary Material**. Further inquiries can be directed to the corresponding author.

## Supplementary material

Supplementary Figure 1 RMSD of the Cα atomic position in the 50 ns trajectory of MD simulations. Left, the Mygalin-IFN-γ complex shows conformational stability starting at 22.5 ns. Right, the trajectory of the Mygalin-TLR3 complex shows conformational stability starting at 11 ns.

Supplementary Figure 2 PCA analysis shows the 20 most important IFN-γ and TLR3 residues that interact with Mygalin. Left, the Mygalin-IFN-γ complex includes Asp164, Pro106, and Ala166, among the most relevant residues identified by molecular docking analysis. Right, the TLR3-Mygalin complex includes Asn141, Lys145, Lys117, Asp116, Gln167, and Asn140, also identified by docking analysis. Asp116 highlight due to its multiple interactions throughout the MD simulation.
