# Supplementary material for: Mygalin reduces inflammation by targeting TLR3 signaling pathway in macrophages
Source: Front Immunol. 2026 Jun 12;17:1729852. doi: 10.3389/fimmu.2026.1729852 (PMC13303013; doi:10.3389/fimmu.2026.1729852)
Supplement: Supplementary Figure 1 — RMSD of the Cα atomic position in the 50 ns trajectory of MD simulations. Left, the Mygalin-IFN-γ complex shows conformational stability starting at 22.5 ns. Right, the trajectory of the Mygalin-TLR3 complex shows conformational stability starting at 11 ns. [file Table1.docx]

**Suplementary material:**

Table 1. TLR3-D binding sites. Sites 1 and 2: interaction with Poly I:C. Site 3: interaction with Mygalin and interface between TLR3-D and TLR3-A.

| **TLR3-D**  **site 1** | **Poly I:C** | **Interaction** |  | **TLR3-D site 2** | **Poly I:C** | **Interaction** |
| --- | --- | --- | --- | --- | --- | --- |
| His39 | inosinic | H bond |  | Arg489 | cytidylic | ionic |
| Lys41 | inosinic | H bond |  | Asn515 | cytidylic | ionic |
| His60 | inosinic | ionic |  | Asn517 | cytidylic | H bond |
| Asn61 | inosinic | H bond |  | Ala519 | inosinic | hydrophobic |
| Gln62 | inosinic | H bond |  | His539 | cytidylic | ionic |
| Arg64 | cytidylic | H bond |  | Asn540 | cytidylic | H bond |
| Arg65 | cytidylic | ionic |  | Asn541 | cytidylic | H bond |
| Phe84 | inosinic | H bond |  | Arg544 | inosinic | H bond |
| Thr86 | cytidylic | H bond / hydrophobic |  | Ser571 | cytidylic | H bond |
| Ser88 | cytidylic | H bond |  | Gly573 | cytidylic | H bond |
| Lys89 | cytidylic | ionic |  | Lys619 | inosinic | H bond |
| Lys108 | inosinic | H bond |  |  |  |  |
|  |  |  |  |  |  |  |
| **TLR3-D**  **sitie 3** | **Mygalin**  **atom (group)** | **Interaction** |  | **TLR3-D**  **site 3** | **TLR3-A** | **Interaction** |
| Ser115 | C (acyl 1) | hydrophobic |  | Ser115 | Asn522 | polar |
| Asp116 | OH (acyl 1) | H Bond |  | Asp116 | Ser498 | polar |
| Asp116 | NH (polyamine) | H Bond |  | Lys117 | Asp523 | polar |
| Asp116 | C (polyamine) | hydrophobic |  | Lys117 | Asp524 | polar |
| Asp116 | OH (acyl 2) | H Bond |  | Lys139 | Ser498 | polar |
| Lys117 | OH (acyl 1) | H Bond |  | Asn140 | Pro499 | hydrophobic |
| Lys117 | C (acyl 1) | hydrophobic |  | Asn141 | Arg473 | polar |
| Asn140 | C (polyamine) | hydrophobic |  | Lys145 | Glu527 | polar |
| Asn141 | O (polyamine) | H Bond |  | Thr166 | Arg473 | hydrophobic |
| Asn141 | C (acyl 2) | hydrophobic |  | Gln167 | Arg47 | polar |
| Val144 | OH (acyl 2) | H Bond |  | Gln167 | Gln503 | polar |
| Val144 | C (acyl 2) | hydrophobic |  |  |  |  |
| Lys145 | OH (acyl 2) | H Bond |  |  |  |  |
| Lys145 | C (acyl 2) | hydrophobic |  |  |  |  |
| Gln167 | O (polyamine) | H Bond |  |  |  |  |
