# Supplementary material for: Mygalin reduces inflammation by targeting TLR3 signaling pathway in macrophages
Source: Front Immunol. 2026 Jun 12;17:1729852. doi: 10.3389/fimmu.2026.1729852 (PMC13303013; doi:10.3389/fimmu.2026.1729852)
Supplement: Supplementary file 2 [file DataSheet1.docx]

**Suplementar Figures 1 and 2 - Author´s Proof 2- Manuscript ID 1729852**


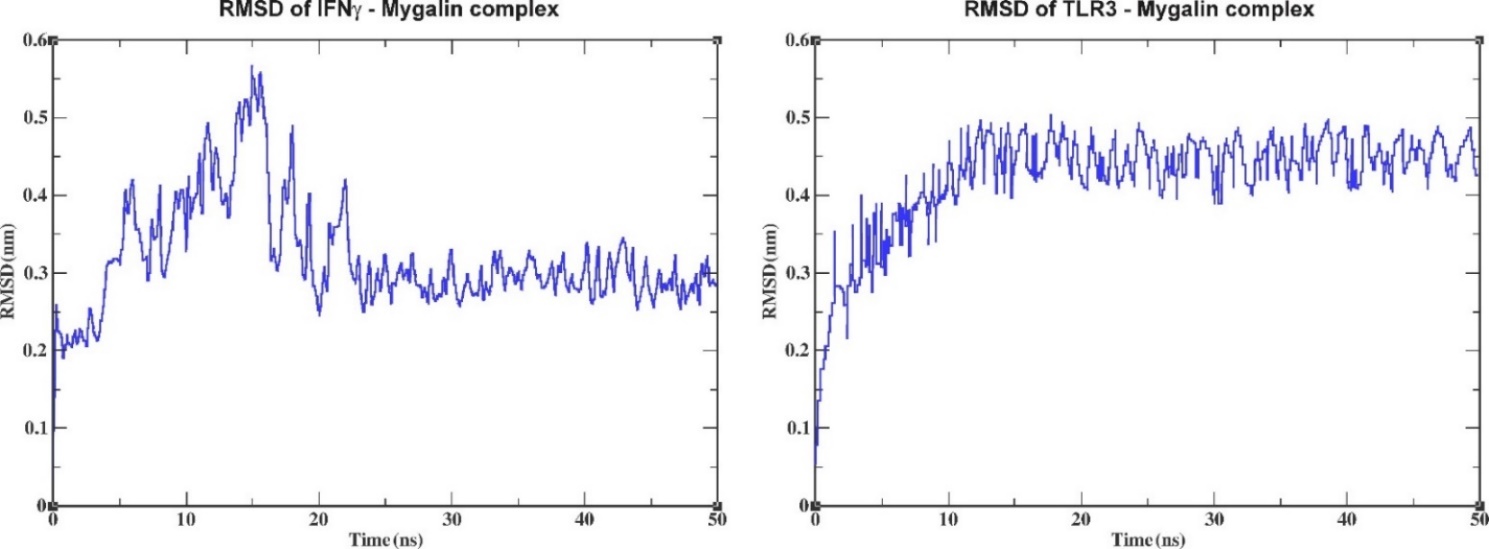


**Supplementary Figure 1**. RMSD of the Cα atomic position in the 50 ns trajectory of MD simulations. Left, the Mygalin-IFN-γ complex shows conformational stability starting at 22.5 ns. Right, the trajectory of the Mygalin-TLR3 complex shows conformational stability starting at 11 ns.


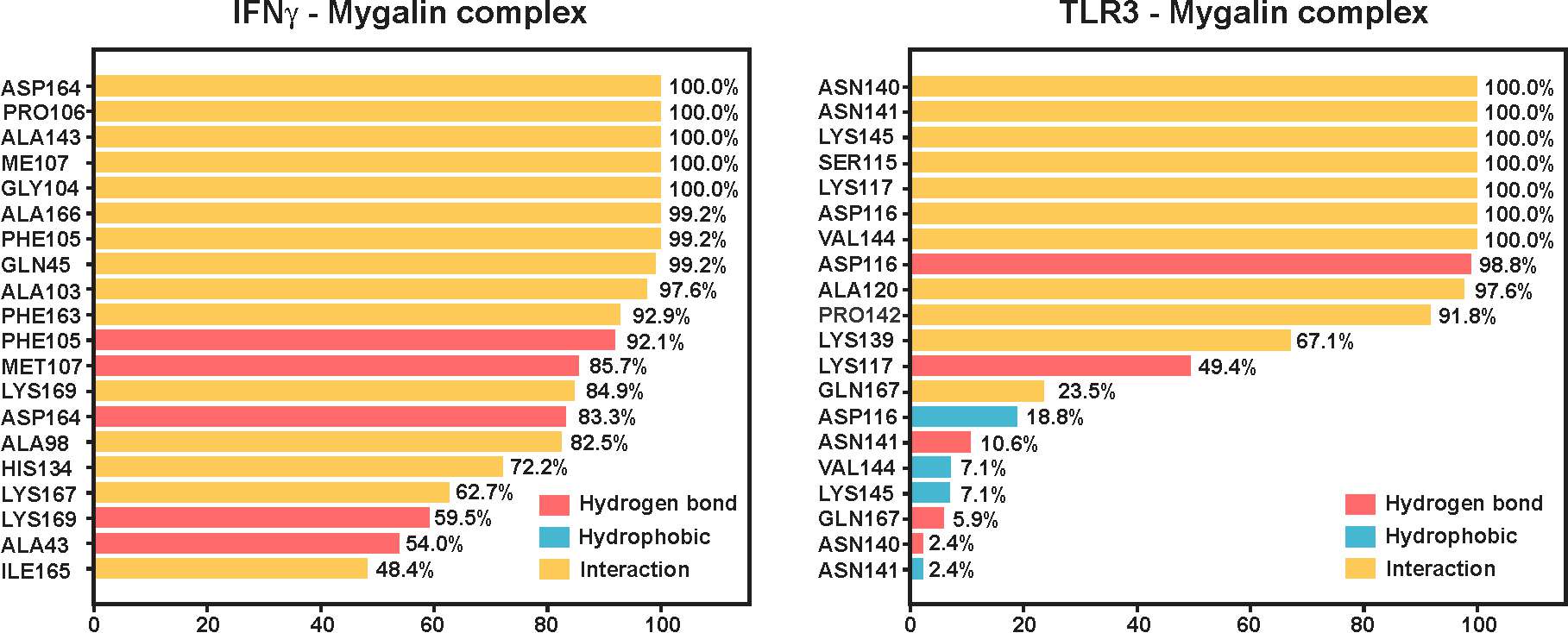


**Supplementary Figure 2**. PCA analysis shows the 20 most important IFN-γ and TLR3 residues that interact with Mygalin. Left, the Mygalin-IFN-γ complex includes Asp164, Pro106, and Ala166, among the most relevant residues identified by molecular docking analysis. Right, the TLR3-Mygalin complex includes Asn141, Lys145, Lys117, Asp116, Gln167, and Asn140, also identified by docking analysis. Asp116 highlight due to its multiple interactions throughout the MD simulation.
